# Therapeutic efficacy of LysGH15 against necrotising pneumonia caused by *Staphylococcus aureus* in a rabbit model

**DOI:** 10.3389/fvets.2025.1529870

**Published:** 2025-02-10

**Authors:** Bowei Zhang, Liran Song, Yongran Wang, Meimei Zhang, Chong Chen, Hui Ning, Li Wang, Cao Qiu, Xinwu Wang, Changjiang Sun, Xin Feng, Wenyu Han, Bin Wang, Yalu Ji, Jingmin Gu

**Affiliations:** ^1^State Key Laboratory for Diagnosis and Treatment of Severe Zoonotic Infectious Diseases, Key Laboratory for Zoonosis Research of the Ministry of Education, Institute of Zoonosis, College of Veterinary Medicine, Jilin University, Changchun, China; ^2^College of Animal Science and Technology, Jilin Agricultural Science and Technology University, Jilin, China; ^3^Jiangsu Co-Innovation Center for the Prevention and Control of Important Animal Infectious Diseases and Zoonoses, Yangzhou University, Yangzhou, China; ^4^Department of Infectious Diseases, Center of Infectious Diseases and Pathogen Biology, Key Laboratory of Organ Regeneration and Transplantation of the Ministry of Education, The First Hospital of Jilin University, Changchun, China

**Keywords:** *Staphylococcus aureus*, lysin, LysGH15, necrotising pneumonia, rabbit

## Abstract

**Introduction:**

*Staphylococcus aureus* (*S. aureus*) is one of the most important zoonotic pathogens and can be transmitted to humans through the meat diet routes, causing necrotising pneumonia.

**Methods:**

This study investigated the therapeutic effect of bacteriophage lysin LysGH15 on necrotising pneumonia in rabbit model caused by *S. aureus*.

**Results:**

In the *in vitro* experiments, 50 μg/mL LysGH15 not only significantly reduced the viable count (approximately 3.24 × 10^6^ CFU/g) of chicken meat stored at 4°C for 48 h but also effectively reduced the viable count of chicken meat thawed at 4°C and 30°C, with reductions of approximately 1.42 × 10^6^ CFU/g and 2.78 × 10^6^ CFU/g, respectively. In the *in vivo* experiments, a single intranasal administration of 300 μg/rabbit increased the survival rate of rabbits to 60%. At 72 h postinfection, the number of bacteria in the lung tissues of the rabbits treated with LysGH15 was 7 × 10^4^ CFU/g, which was significantly lower than that in the lung tissues of rabbits treated with PBS (7.76 × 10^6^ CFU/g) or linezolid (6.38 × 10^5^ CFU/g). In addition, LysGH15 treatment alleviated lung tissue damage in infected rabbits and significantly reduced the levels of Panton-Valentine leukocidin (PVL), alpha-toxin (Hla), and the cytokines IFN-*γ*, TNF-*α*, and IL-8 in their lung tissues, similar to those in rabbits treated with linezolid.

**Discussion:**

These results suggest that LysGH15 has the potential to be used as a novel antimicrobial agent for the treatment of necrotising pneumonia caused by *S. aureus*.

## 1 Introduction

*Staphylococcus aureus* (*S. aureus*) is a Gram-positive bacterium ubiquitous in the natural world and an important zoonotic and foodborne pathogen (1). Among many infectious diseases, pneumonia caused by *S. aureus* is one of the most serious infections and is associated with high mortality, especially in patients with fulminant necrotising pneumonia (2). Some antibiotics, such as linezolid, have been shown to have therapeutic effects on necrotising pneumonia in rabbit model. However, the problem of antibiotic resistance is becoming more serious, and the use of antibiotics in human is limited (3). Therefore, there is an urgent need to find alternative therapies to antibiotics.

*Staphylococcus aureus* can be transmitted to humans through various pathways, one of which is through food transmission (4). According to statistics, food poisoning incidents caused by *S. aureus* account for approximately 25% of total foodborne microbial food poisoning incidents (5). Most staphylococcal and enterotoxin contamination in meat products is due mainly to cross-contamination during production or raw meat storage, which allows pathogenic staphylococci to produce enough enterotoxins to cause disease (6). With increasingly strict regulations on meat additives by government departments worldwide, conventional antibacterial agents with high bactericidal effects but pose potential threats to human health can no longer be used for microbial control in chicken products (7). Identifying a safe and effective new antimicrobial agent to eliminate *S. aureus* in meat products has become a research hotspot.

Phage lysin is a cell wall hydrolytic enzyme synthesized by the phage gene coding in the later stage of phage infection of bacteria (8). Lysin can target peptidoglycan in bacterial cell walls, causing peptidoglycan lysis and resulting in bacterial cell wall rupture, leading to bacterial death (9). Compared with traditional antibiotic therapy, lysin has many advantages. For example, the unique mode of action of lysin avoids the resistance mechanism of antibiotics and is equally effective against antibiotic-resistant strains (10). In addition, lysin only targets specific bacterial groups, reducing collateral damage to surrounding microbial communities (11). Lysin therapy has been validated in various animal models, and no toxic side effects have been observed (8, 10).

Several studies have explored the preventive and therapeutic effects of antibodies and antibiotics on *S. aureus* induced necrotizing pneumonia using a rabbit model (12–16). However, there is currently no research on the therapeutic effect of phage lysin on necrotising pneumonia. Our previous studies have indicated LysGH15, a lysin encoded by the phage GH15, displays efficient lytic activity against MRSA strains and antibiotics sensitive *S. aureus* strains isolated from the clinics (17). In this study, we investigated the bactericidal effect of the phage lysin LysGH15 as a novel bactericidal agent in meat under different storage and thawing conditions and evaluated the therapeutic efficacy of LysGH15 on necrotising lungs caused by *S. aureus* in rabbit model.

## 2 Materials and methods

### 2.1 Ethical statements

All the animal studies were approved by the Animal Welfare and Research Ethics Committee at Jilin University (Permit Number: SY202309034). This study adheres to the Animal Research: Reporting of *In Vivo* Experiments (ARRIVE) guidelines. All methods were conducted in strict accordance with the relevant guidelines and regulations. The animals were treated humanely, and every effort was made to minimize suffering.

### 2.2 Animals

Female New Zealand white rabbits weighing 1.5–1.8 kg was purchased from the Experimental Animal Centre of Jilin University. The rabbits were housed in cages in a temperature-controlled animal room with a 12 h light/dark cycle. Feed and fresh water were available ad libitum.

### 2.3 Bacterial strain and culture conditions

The *S. aureus* S6 used in this study were isolated from the lung tissues of rabbits suffering from necrotising pneumonia and has been identified as a multidrug-resistant bacterium (18). *S. aureus* was routinely grown in brain heart infusion (BHI) broth (Becton, Dickinson and Company, USA) at 37°C in the shaker (ISS-7100, Lab Companion, Jeio Tech Co., Ltd., Korea) with shaking at 200 rpm.

### 2.4 Preparation of LysGH15

In this study, an *Escherichia coli* BL21 (DE3) competent cell expressing the full-length LysGH15 protein was constructed, and protein purification was performed according to a previous study (17). The purity and molecular weight of the recombinant proteins were detected via sodium dodecyl sulfate-polyacrylamide gel electrophoresis (SDS–PAGE).

### 2.5 The bactericidal activity assay of LysGH15 in BHI medium

*Staphylococcus aureus* S6 was cultured in BHI medium at 37°C and 180 rpm under oscillation and subsequently centrifuged to collect the bacteria in the logarithmic growth phase (OD_600_ = 0.6). The bacteria were washed three times with sterile phosphate-buffered saline (PBS) after centrifugation (8,000 × *g*, 5 min), and purified LysGH15 was added to the suspension of *S. aureus* S6 to final concentrations of 20, 50, and 100 μg/mL. The mixture was incubated at 37°C for 30 min, after which the CFU values were determined.

### 2.6 Determination of the bactericidal activity of LysGH15 in chicken meat

Raw chicken breast meat was purchased from a local retail store in Changchun (China) on October 8, 2023. Fresh chicken breast was treated according to the methods described (19). Specifically, fresh chicken breast tissue was cut into small pieces of 1 × 1 cm^2^ (approximately 1 g) and irradiated for 30 min on both the front and back sides at a distance of 0.3 m from the UV source for sterilization. One hundred microliters of *S. aureus* S6 solution (approximately 1 × 10^8^ CFU/mL) that cultured to logarithmic growth phase (OD_600_ = 0.6) was evenly titrated on the surface of the meat for 15 min so that *S. aureus* was completely adhered to the meat. Then, 100 μL of different concentrations of LysGH15 (10 μg/mL, 50 μg/mL, or 100 μg/mL) and sterile PBS (pH = 7.0) were dripped onto the surface of the meat. To simulate thawing under normal conditions, the meat was stored at −20°C for 24 h and then thawed at 4°C. The five groups of chicken meat were collected after 0, 2, 6, 24, and 48 h. To simulate thawing under abnormal conditions, the meat was stored at −20°C for 24 h and then thawed at 30°C, and the five groups of chicken meat were collected after 0, 1, 2, 3, and 4 h. To simulate the preservation of fresh chicken meat, the meat was stored at 4°C, and the four groups of chicken meat were collected at 0, 3, 24, and 48 h. The collected chicken meat from the four groups was homogenized in 1 mL of PBS. The bacterial load was determined via *S. aureus* selective medium, and the results were calculated and expressed as CFU/g.

### 2.7 Evaluation of the therapeutic effect of LysGH15 in rabbits with necrotising pneumonia

A rabbit necrotising pneumonia model was established based on a previous study (20, 21). Briefly, New Zealand white rabbits were anaesthetized with ketamine hydrochloride (35 mg/kg, i.m.) and xylazine hydrochloride (5 mg/kg, i.m.) (22) and intranasally injected with 1 mL of a single dose of *S. aureus* S6 (3 × 10^8^ CFU/per rabbit) into the lungs.

To evaluate the protection rate of LysGH15 in infected rabbits, rabbits infected with *S. aureus* S6 were randomly divided into five groups. Three groups received an intranasal drop of 1 mL of LysGH15 (100 μg, 200 μg, or 300 μg/per rabbit) 1 h after infection; one group received an intramuscular injection of 50 mg/kg linezolid 1 h after infection; and one group received an intranasal drop of 1 mL of sterile PBS buffer 1 h after infection as a control. There were 10 rabbits in each group. The number of dead rabbits was recorded every 24 h during the 7-day observation period.

In a separate subsequent second experiment, mitigating effect of LysGH15 were studied on pathological parameters in lung tissues and blood of rabbits with induced necrotizing pneumonia, and the therapeutic dose of 300 μg/per rabbit of LysGH15 was used for evaluation. The rabbits were randomly divided into four groups: (i) rabbits treated with 1 mL of sterile PBS buffer by intranasal drip 1 h after infection; (ii) rabbits treated with a single dose of LysGH15 (1 mL, 300 μg) by intranasal drip 1 h after infection; (iii) rabbits intramuscularly injected with 50 mg/kg linezolid 1 h after infection; and (iv) rabbits without any treatment served as the control group.

Determination of the bacterial load in rabbit lung tissues. Three rabbits from each group were euthanized by carbon dioxide asphyxiation (22) at 24 and 72 h postinfection. Lung tissues (1–2 cm) were weighed and homogenized in 1 mL of PBS. Bacterial loads were determined on dilutions of the ground lung tissue.

Histopathologic analysis. Three rabbits from each group were euthanized by carbon dioxide asphyxiation at 24 and 72 h postinfection. The lungs of the rabbits were removed and immediately placed in 4% formalin. The formalin-fixed tissues were stained with hematoxylin and eosin and analyzed for changes in the histopathological characteristics of the lungs via microscopic observation.

Determination of cytokines and toxins in rabbit blood and lung tissue. The levels of cytokines in the blood and lung tissue and the Panton-Valentine leukocidal leukocytocin (PVL) and alpha toxin (Hla) levels in the lung tissue from the different groups were measured. Briefly, three rabbits from each group were euthanized by carbon dioxide asphyxiation at 24 and 72 h postinfection. The cardiac blood of each rabbit was collected, immediately placed in a 37°C incubator for 1 h and then placed at 4°C to separate the serum. In addition, 1–2 cm of lung tissue was weighed, suspended in sterile PBS and homogenized in 1 mL of PBS. The levels of the cytokines IL-8, TNF-*α* and IFN-*γ* in the blood and ground lung tissue samples and the levels of PVL and Hla in the ground lung tissue samples were determined via rabbit-specific enzyme-linked immunosorbent assay (ELISA) kits (USCN Life Science Inc., Wuhan, China) (23, 24).

### 2.8 Statistical analysis

SPSS version 13.0 software (SPSS, Inc., Chicago, IL, United States) was used for all the statistical analyses. All the experimental data were analyzed via one-way analysis of variance. *p* < 0.05 was considered a difference, and *p* < 0.01 was considered significant. *p* < 0.001 was considered extremely significant. The error bars represent the standard deviation.

## 3 Results

### 3.1 Efficiency of LysGH15 in the reduction of *S. aureus* in BHI liquid culture medium and raw chicken meat

**Figure 1:**
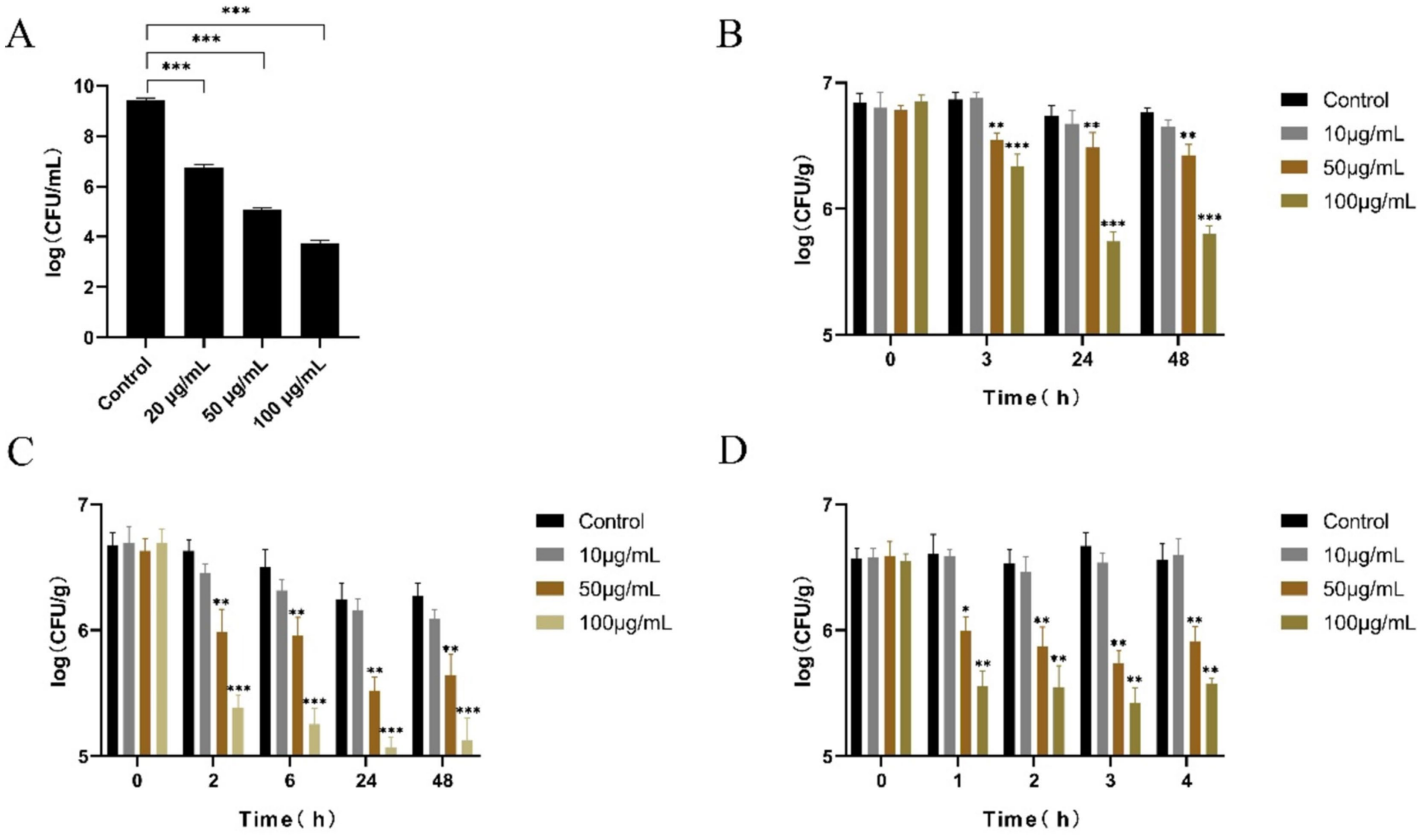
The bactericidal activity of LyGH15 against *Staphylococcus aureus* in BHI medium and chicken meat under different storage conditions. **(A)** Determination of the bactericidal activity of different concentrations of LysGH15 against *S. aureus* in BHI medium. **(B)** LyGH15 and chicken meat were stored at 4°C, and the bacterial load in the chicken meat was measured at different time points. **(C)** LyGH15 and chicken meat were stored at −20°C for 24 h and then thawed at 4°C, after which the bacterial load in the chicken meat was measured at different time points after thawing. **(D)** LyGH15 and chicken meat were stored at −20°C for 24 h and then thawed at 30°C, after which the bacterial load in the chicken meat was measured at different time points after thawing. The data represent the means ± standard deviations (SDs) of triplicate experiments. *p < 0.05, **p < 0.01, ***p < 0.001.

As shown Figure 1A, the bactericidal activity of LysGH15 against *S. aureus* S6 was dose-dependent in BHI liquid culture medium, and 20, 50, and 100 μg/mL LysGH15 killed approximately 3 lg, 4 lg, and 6 lg units of *S. aureus* S6 within 30 min, respectively. When LyGH15 and chicken meat were stored at 4°C for 3 h, 50 μg/mL LysGH15 significantly reduced the amount of *S. aureus* (approximately 3.24 × 10^6^ CFU/g) compared with that in the control group (*p* < 0.01) (Figure 1B). When LyGH15 and chicken meat were stored at −20°C for 24 h, 50 μg/mL LysGH15 significantly reduced the number of *S. aureus* (approximately 1.42 × 10^6^ CFU/g) after thawing at 4°C for 2 h (*p* < 0.01) (Figure 1C). In addition, when LyGH15 and chicken meat were stored at −20°C for 24 h, 50 μg/mL LysGH15 significantly reduced the number of *S. aureus* (approximately 2.78 × 10^6^ CFU/g) after thawing at 30°C for 1 h (*p* < 0.05) (Figure 1D).

### 3.2 LysGH15 improved the survival outcome of rabbits with necrotising pneumonia

**Figure 2:**
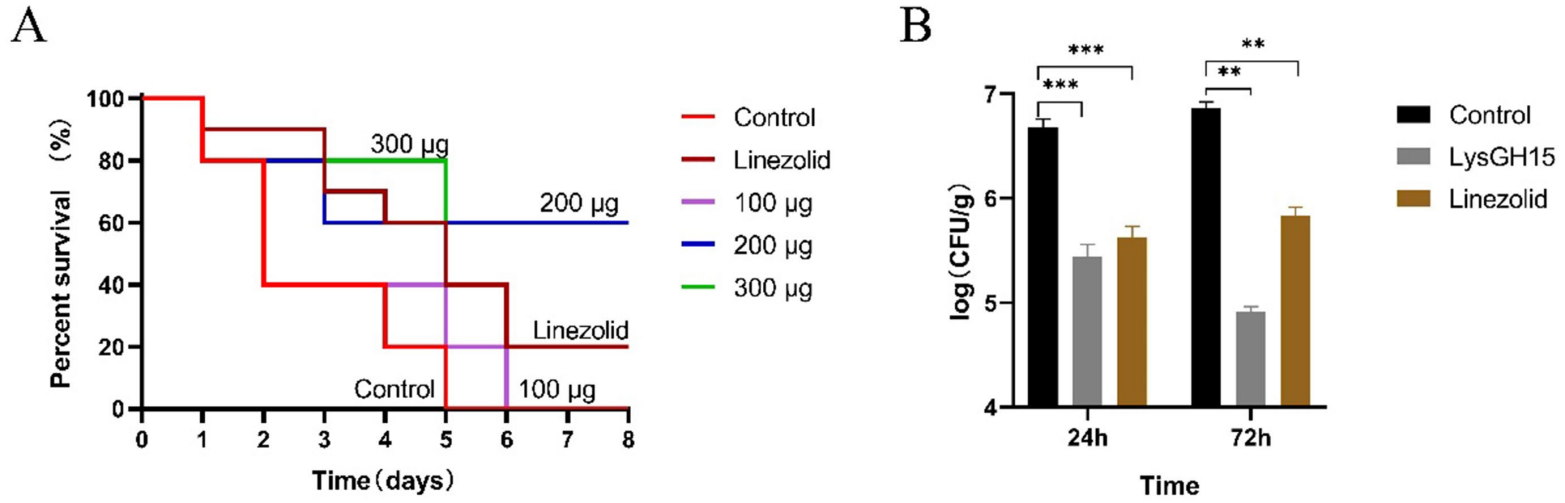
LysGH15 improved the survival outcome of rabbits infected with a lethal dose of *S. aureus* S6. **(A)** Survival rates. The rabbits were intranasally challenged with 3 × 10^8^ CFU of *S. aureus* S6, and 1 h later, the challenged rabbits were intranasally treated with different doses of LysGH15. The control rabbits were treated with linezolid or PBS under the same conditions. Each group contained 10 rabbits. **(B)** Colony counts from the rabbit lung samples. Each group contained three rabbits. The lungs of the rabbits were harvested and homogenized at 24 h and 72 h after infection with *S. aureus* S6. Each bar represents the average count of three rabbits. The values represent the means and standard deviations. **p < 0.01, ***p < 0.001.

As shown Figure 2A, in the control group, a single dose of 3 × 10^8^ CFU/per rabbit of *S. aureus* resulted in a 60% (6/10) mortality rate within 2 days and a 100% (10/10) mortality rate within 5 days. Compared with the control group, the LysGH15-treated group presented prolonged survival time and an increased final survival rate (Figure 2A). On day 5 postinfection, a single dose of 100 μg/per rabbit of LysGH15 increased the survival rate of the rabbits to 20% (2/10). A single dose of 200 μg or 300 μg/per rabbit of LysGH15 increased the survival rate of the rabbits to 60% (6/10) and 80% (8/10), respectively, and the survival rate was ultimately maintained at 60% (6/10). In addition, the linezolid (50 mg/kg) treatment increased the survival rate of rabbits to 40% (4/10) on day 5 postinfection, and the survival rate was ultimately maintained at 20% (2/10).

### 3.3 LysGH15 improved the lung tissue of rabbits suffering from necrotising pneumonia

At 24 and 72 h postinfection, the bacterial counts in the lung tissues of the control rabbits reached approximately 4.36 × 10^6^ CFU/g and 7.76 × 10^6^ CFU/g, respectively (Figure 2B). In contrast, a single dose of 300 μg of LysGH15 significantly reduced the bacterial load in rabbit lung tissues, with rabbit lung tissue bacterial counts reaching approximately 2.92 × 10^5^ CFU/g and 7.11 × 10^4^ CFU/g at 24 h and 72 h postinfection, respectively. In the linezolid treatment group, the rabbit lung tissue bacterial counts reached approximately 4.44 × 10^5^ CFU/g and 6.38 × 10^5^ CFU/g at 24 and 72 h postinfection, respectively (Figure 2B).

**Figure 3:**
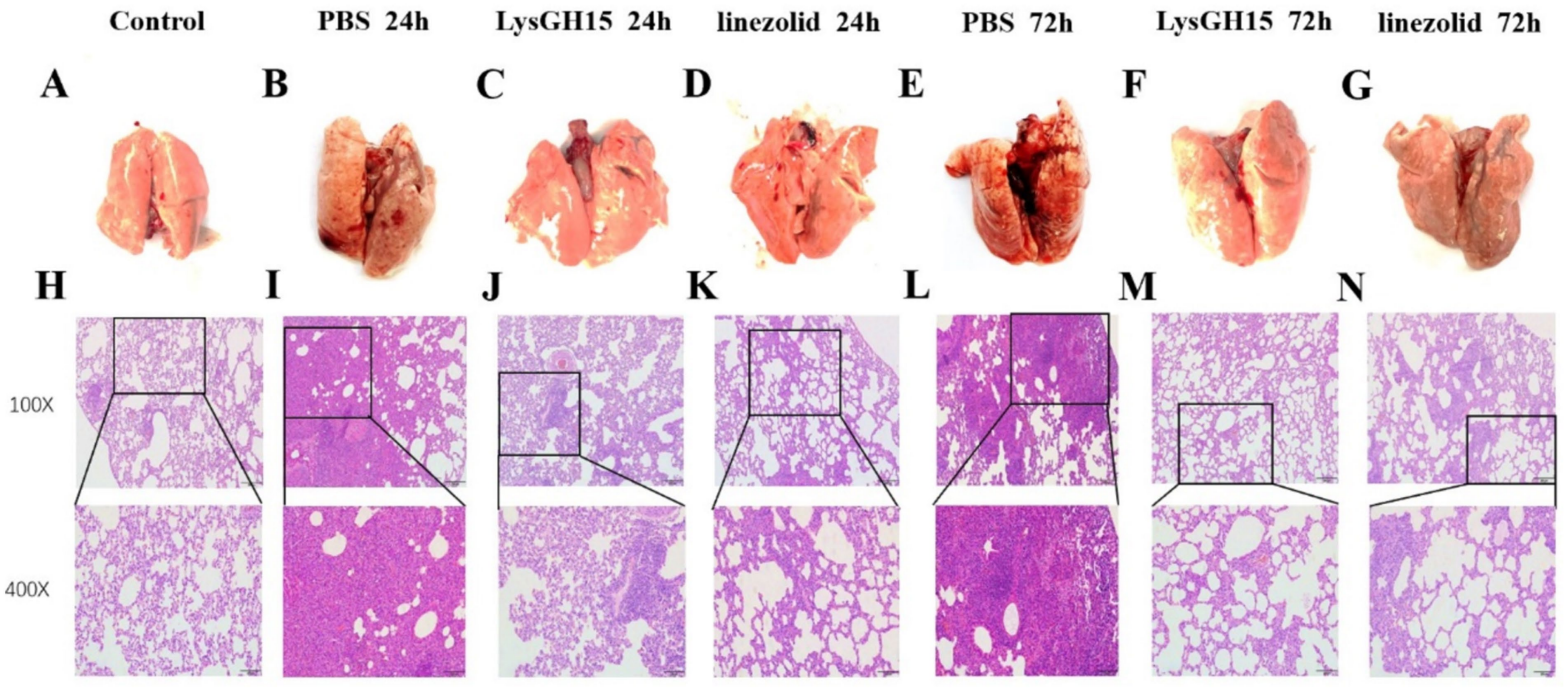
Gross pathology and histopathology of the lung tissue. At 24 h and 72 h postinfection, the lungs were removed from the rabbits treated with 300 μgLysGH15, linezolid or PBS. The lungs of healthy rabbits were used as controls **(A-G)**. The tissue samples were stained with hematoxylin and eosin **(H-N)**.

LysGH15 significantly improved the pathological damage caused by *S. aureus* to rabbit lungs. On the surface, the lung tissue from the control rabbits was pink, and the surface was rosy and smooth (Figure 3A). However, the lungs of the rabbits infected with *S. aureus* were dark red or even purple-black (Figures 3B,E). In contrast, the lungs of the rabbits in the LysGH15 treatment group and antibiotic treatment group had similar appearances, with a smooth surface and pink color 24 h after treatment (Figures 3C,D) and a dark red color in the local area 48 h after treatment (Figures 3F,G). According to the pathological analysis, the alveolar structure of the control lung tissue was clear, and there was no inflammatory cell infiltration in the alveolar spaces (Figure 3H). In contrast, after infection with *S. aureus*, the rabbit lung membranes were thickened, the alveolar intervals were thickened, capillary dilatation and congestion of the alveolar walls occurred, and the alveolar epithelium was necrotic and exfoliated, accompanied by a large amount of inflammatory cell infiltration (Figures 3I,L). In contrast, inflammation and pathological changes were significantly alleviated in the lung tissue of the rabbits that were treated with lysin LysGH15, and similar results were observed in the linezolid treatment group (Figures 3J,K,M,N).

**Figure 4:**
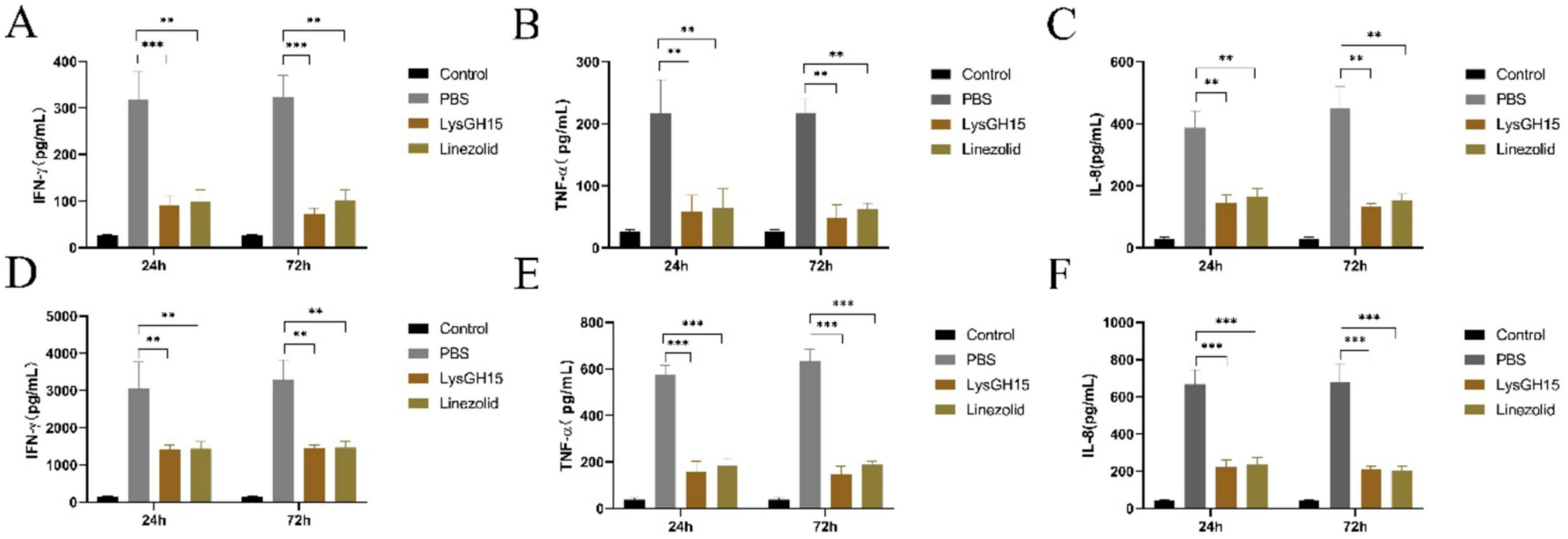
Cytokine levels in the blood and lung tissues. **(A–C)** Determination of the levels of the cytokines IFN-*γ*, TNF-*α*, and IL-8 in the blood. **(D–F)** Determination of the levels of the cytokines IFN-γ, TNF-α, and IL-8 in the lung tissue homogenate. Each group contained 3 rabbits. **p < 0.01, ***p < 0.001.

**Figure 5:**
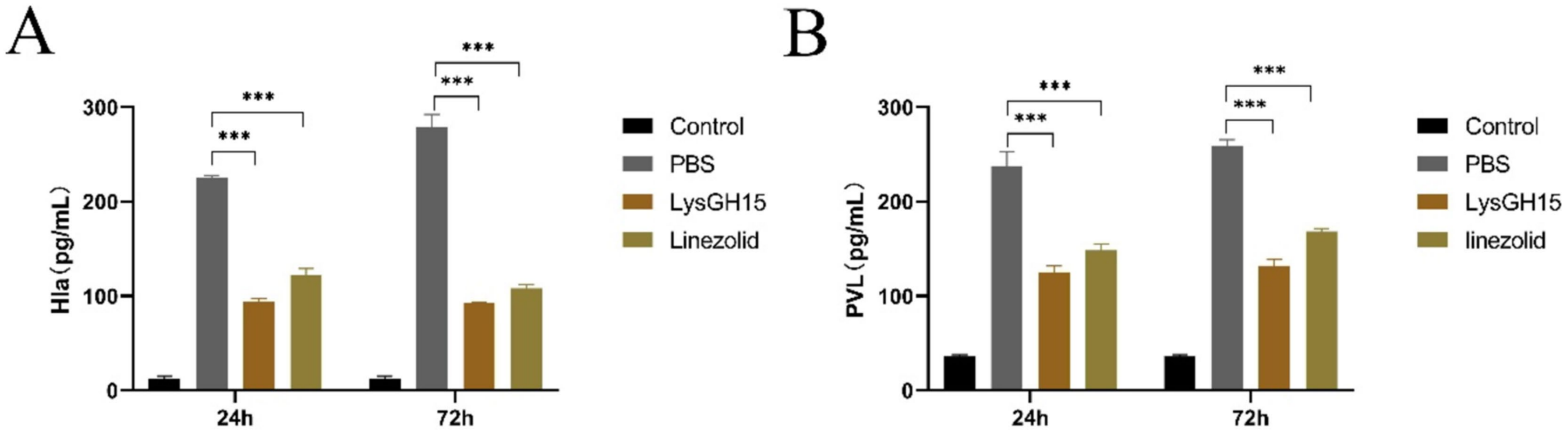
Toxin levels in the lung tissues. Determination of the PVL **(A)** and Hla **(B)** toxin concentration in the lung tissue homogenate. The lungs of the healthy rabbits were used as controls. Each group contained 3 rabbits. ***p < 0.001.

In addition, several key inflammatory cytokines and toxins in the blood and lung tissues of rabbits in each group were measured. As shown in Figure 4, the levels of the cytokines IFN-*γ*, TNF-*α*, and IL-8 in the blood and lung tissues were significantly greater in the infected rabbits than in the control rabbits at 24 and 72 h postinfection. Compared with the PBS treatment, LysGH15 treatment also significantly reduced the cytokine IFN-γ, TNF-α and IL-8 in the blood (Figures 4A–C) and lung tissues (Figures 4D–F) of rabbits. In addition, LysGH15 treatment significantly reduced the levels of toxins PVL and Hla in the lung tissues of rabbits (Figures 5A,B). These decreases were similar to the linezolid treatment group (Figures 4, 5).

## 4 Discussion

As an important zoonotic pathogen, the emergence and spread of multidrug-resistant *S. aureus* in different environments continue to pose significant challenges to its prevention and treatment in humans (25, 26). Although antibiotics linezolid and Tedizolid have improved necrotising pneumonia caused by *S. aureus* in rabbit model, the improvement effect needs to be further improved (20). Previous studies have shown that bacteriophages have a better protective effect against *S. aureus* necrotic pneumonia in rabbits than the antibiotic linezolid (18). In comparison, the lysins encoded by phage has greater bactericidal efficiency than phage, which is attributed to the fact that lysins can directly act on the peptidoglycan scaffold of the cell wall from the outside, hydrolyzing peptidoglycan to kill bacteria (27). Due to the lack of outer membrane protection in the cell wall of Gram-positive bacteria, lysins are particularly effective in Gram-positive bacteria, leading to osmotic shock and cell rupture, thereby achieving the goal of rapidly killing pathogens (28). Therefore, the use of phage lysins is more promising, by enabling the development of broad host range potent antimicrobials (29, 30).

Although there have been numerous studies on phage lysins in animal models of bacterial infection, there is currently no research on the treatment of lysins in rabbit models of necrotizing pneumonia. In this study, we evaluated the therapeutic effect of *S. aureus* phage lysin on necrotic pneumonia in rabbits. Because *S. aureus* can rapidly cause necrotising pneumonia in rabbits (21), combined with previous studies on phage therapy (18), we chose to treat infected rabbits with a single dose of LysGH15 administrated 1 h after infection. Encouragingly, the protective effect of single-dose LysGH15 treatment on infected rabbits was superior to that of antibiotics. Lysin can rapidly act and lyse bacteria (31), in contrast, most antibiotics, including linezolid, inhibit basic bacterial metabolic steps, leading to slow deterioration of cellular conditions and ultimately cell death (31). Therefore, antibiotics take a long time to kill bacteria. This is a potential explanation for the superiority of lysins over linezolid in reducing bacterial load and improving survival outcomes.

*Staphylococcal pneumonia* is largely driven by Hla and PVL (20, 32). In this study, treatment with 300 μg/rabbit lysin LyGH15 significantly reduced the levels of the two toxins in infected rabbit lung tissue at 24 and 72 h post-treatment. However, after LyGH15 treatment, 40% of the infected rabbits still died one after another, which may be due to the lethal damage caused by the toxins PVL and Hla to the rabbits at the beginning of the treatment window. Research has shown that attenuated forms of Hla or PVL alone can prevent fatal pneumonia in only partially infected rabbits (32). Therefore, combining lysin with vaccines targeting Hla and PVL toxins may provide better protection against necrotising pneumonia caused by *S. aureus*.

Due to the fact that *S. aureus* can be transmitted to humans through meat products, this study also explored the sterilization effect of LyGH15 on *S. aureus* in frozen chicken meat during thawing under recommended and temperature abuse conditions. After processing raw chicken meat, storing it at a refrigerated temperature is recommended (19). In this study, the lysin LyGH15 significantly reduced the number of *S. aureus* in chicken meat during refrigeration. In addition, frozen storage is a method of maintaining meat quality and controlling the growth of bacterial pathogens. *S. aureus* can survive under such harsh conditions (33), which is consistent with our findings (Figures 1C,D). Usually, frozen meat is thawed at 4°C. If frozen meat is not properly handled, such as when left on a countertop, rising temperatures will rapidly breed pathogenic bacteria (19, 34). However, our research results indicate that, in chicken meat contaminated with *S. aureus*, whether thawed at 4°C or a high temperature of 30°C, LyGH15 can effectively reduce the number of *S. aureus* in chicken meat.

The activity of phage and lysins against pathogenic bacteria has been confirmed in food raw materials and specific foods, which clearly indicates that they can be used to combat foodborne pathogens in the food sector (35). In addition, phage have become commercially available products in some cases, which can eliminate pathogenic bacteria in livestock and various food matrices (36). However, the application of lysins in food protection has not been fully explored. The important difference between lysins and phage is that lysins have a lower likelihood of developing drug resistance, while phage is unique in their autodosing and evolution capacity (37). Therefore, with the continuous increase and deepening of lysins in clinical research, engineered phage lysins are expected to become an effective tool for combating foodborne pathogens.

## 5 Conclusion

In this study, the results of bactericidal activity experiments on chicken meat revealed that LysGH15 could effectively control the contamination of *S. aureus* in meat products under chilled and thawed conditions. *In vivo* experiments revealed that LysGH15 treatment effectively reduced the number of bacteria in infected rabbit lungs, inhibited the production of bacterial toxins, reduced the production of cytokines, significantly improved the pathological manifestations of lung tissues, and ultimately increased the survival rate. These results suggest that LysGH15 has the potential to be used as a novel antimicrobial agent for controlling the growth of *S. aureus* in chilled and thawed chicken meat and for the treatment of necrotising pneumonia caused by *S. aureus*.

## Data Availability

The datasets presented in this study can be found in online repositories. The names of the repository/repositories and accession number(s) can be found at: https://www.ncbi.nlm.nih.gov/genbank/, HM015284.
